# Antidiabetic Properties of Curcumin II: Evidence from In Vivo Studies

**DOI:** 10.3390/nu12010058

**Published:** 2019-12-25

**Authors:** Danja J. Den Hartogh, Alessandra Gabriel, Evangelia Tsiani

**Affiliations:** 1 Department of Health Sciences, Brock University, St. Catharines, ON L2S 3A1, Canada; dd11qv@brocku.ca (D.J.D.H.); ag15kh@brocku.ca (A.G.); 2 Centre for Bone and Muscle Health, Brock University, St. Catharines, ON L2S 3A1, Canada

**Keywords:** insulin resistance, diabetes, curcumin, curcuminoids, in vivo, animal studies, human studies

## Abstract

Type 2 diabetes mellitus (T2DM) is a growing metabolic disease characterized by insulin resistance and hyperglycemia. Current preventative and treatment approaches to insulin resistance and T2DM lack in efficacy, resulting in the need for new approaches to prevent and treat the disease. In recent years, epidemiological studies have suggested that diets rich in fruits and vegetables have beneficial health effects, including protection against insulin resistance and T2DM. Curcumin, a polyphenol found in turmeric, and curcuminoids have been reported to have antioxidant, anti-inflammatory, hepatoprotective, nephroprotective, neuroprotective, immunomodulatory and antidiabetic properties. The current review (II of II) summarizes the existing in vivo studies examining the antidiabetic effects of curcumin.

## 1. Introduction

Insulin resistance is characterized by a reduction in the responsiveness of target tissues to the normal circulating levels of insulin [1,2,3]. Insulin resistance and T2DM are associated with inflammation, obesity, aging and a sedentary lifestyle, and results in hyperglycemia, a state of elevated plasma glucose levels [1,2,3,4,5,6,7]. Hyperglycemia can lead to long-term complications including macrovascular and microvascular damage, cardiovascular disease, retinopathy, neuropathy and nephropathy [1,2,3,4,5,6,7].

Epidemiological studies have suggested that diets rich in fruit and vegetables help regulate body weight (obesity) and protect against cardiovascular disease, cancer and diabetes [8,9,10]. However, it is difficult to determine the role of food components in disease prevention and treatment. Specific components, known as polyphenols, have gained attention within the scientific community for their potential health benefits and preventative and therapeutic properties against chronic diseases [11,12,13,14,15,16].

Polyphenols have been established to have antioxidant properties [17] and to possess a variety of other biological effects such as regulating enzymes [18,19,20]. Therefore, they may prevent diseases through mechanisms that are both dependent and independent of their antioxidant properties [18,19,20].

**Figure 1:**
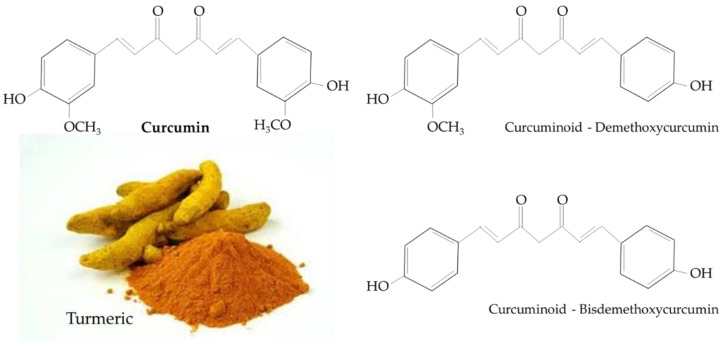
Chemical structure of curcumin and curcuminoids found in turmeric.

Turmeric is a rhizomatous medicinal perennial plant (*Curcuma longa*) and has a rich history of being used in Asian countries, such as China and South East Asia [21,22]. The main natural polyphenol in *C. longa* and in other *Curcuma* species is known as either curcumin (1,7-bis(4-hydroxy-3-methoxyphenyl)-1,6-heptadiene-3,5-dione), or diferuloylmethane [22]. Other curcuminoids, such as demethoxycurcumin and bisdemethoxycurcumin, are structurally similar to curcumin and differ only with respect to the number of methoxy groups on the aromatic rings (Figure 1) [22].

Despite the reported benefits of curcumin by its anti-inflammatory and antioxidant mechanisms, curcumin’s poor bioavailability caused by its poor absorption, rapid metabolism and rapid elimination, limits its potential [23]. Further research is needed to examine the bioavailability and pharmacokinetics of curcumin.

A previous review by Meng et al. in 2013 [24] summarizing the effects of curcumin against different diabetic complications, such as vasculopathy and nephropathy, focused mainly on the antioxidant and anti-inflammatory properties. We have searched the scientific literature focusing on the studies investigating the antidiabetic properties of curcumin. We have summarized all the available information, including the recent studies after 2013, and presented it in two review manuscripts. The first manuscript (Antidiabetic properties of curcumin I: Evidence from in vitro studies) focuses on the in vitro evidence (accepted for publication in *Nutrients*). The second manuscript (Antidiabetic properties of curcumin II: Evidence from in vivo studies) is the current review and focuses on the in vivo evidence. All existing in vivo animal and human studies are presented, where the key words: curcumin, curcuminoid, STZ, alloxan, high-fat diet, high-sugar diet, obesity and diabetes were used in a PubMed search. These key words were searched in multiple different combinations to ensure that all existing in vivo animal and human studies were included. The studies are presented chronologically, and in addition to the text, are organized and presented as a table to give the reader easier access to the information.

## 2. Antidiabetic Effects of Curcumin: In Vivo Animal Studies

### 2.1. Streptozotocin (STZ)-Induced Diabetes Model

The effects of curcumin were examined in a few studies using streptozotocin (STZ)-induced diabetes animal models, and have been summarized in Table 1. T2DM was induced in vivo in Wistar rats by a single intraperitoneal (i.p.) injection of STZ (60 mg/kg b.w.) in the study by Babu and Srinivasan [24] followed by a diet supplementation of curcumin (0.5% diet) for 8 weeks [25]. Curcumin supplementation significantly prevented renal lesion progression and damage. In addition, urinary enzyme (alanine aminotransferase (ALT), alkaline phosphatase (ALP), aspartate aminotransferase (AST) and acid phosphatase) levels were reduced with curcumin supplementation. Curcumin supplementation reduced renal glucose-6-phosphatase and lactate dehydrogenase and increased ATPase activities [25]. The polyunsaturated fatty acid/saturated fatty acid (PUFA/SFA) ratio was increased, while blood triglyceride and phospholipid levels were reduced with curcumin supplementation. Overall, curcumin supplementation reduced diabetic nephropathy [25].

Administration of STZ-induced diabetic Wistar rats with curcumin (300 mg/kg b.w./day) for 8 weeks resulted in significantly reduced fasting plasma glucose, cholesterol, triglyceride and low-density lipoprotein (LDL)-cholesterol levels [26]. 

This was accompanied with increased plasma protein and creatinine levels and decreased plasma urea levels, indicating improved kidney function. Curcumin administration also exerted antioxidant effects with reduced lipid peroxidation and increased superoxide dismutase (SOD) and catalase activities [26].

In the study by Murugan and Pari [27], oral administration of tetrahydrocurcumin (THC; 80 mg/kg b.w./day), a metabolite of curcumin, or curcumin (80 mg/kg b.w./day) to STZ-induced diabetic Wistar rats for 45 days, significantly reduced plasma glucose and increased plasma insulin levels [27]. THC and curcumin administration protected against lipid peroxidation with increased SOD, catalase, glutathione peroxidase (GPx) and glutathione-S-transferase activities, and reduced thiobarbituric acid-reactive substances (TBARS) and hydrogen peroxide (H_2_O_2_) levels in diabetic liver and kidneys [27].

Administration of STZ-induced, diabetic, Sprague–Dawley rats with curcumin (15 and 30 mg/kg b.w./day) for 2 weeks resulted in significantly attenuated renal dysfunction and oxidative stress [28]. Serum glucose and creatinine levels and urine albumin were reduced, urine creatinine clearance was increased with curcumin administration. In addition, curcumin administration reduced oxidative stress with reduced lipid peroxidation and malondialdehyde (MDA) levels, while antioxidant glutathione levels and SOD and catalase activities were increased in kidney tissue [28]. Kidney morphology was improved with curcumin administration, with reduced glomerular thickening, interstitial fibrosis and arteriolopathy. These data suggest that curcumin administration exerts antioxidant effects to attenuate diabetes-induced renal dysfunction and oxidative stress [28].

In a study by Suryanarayana et al. [29], administration of STZ-induced diabetic Wistar-NIN rats with curcumin (0.01% diet) for 8 weeks resulted in reduced lipid peroxidation and oxidative stress [29]. Curcumin administration reduced heart, liver, kidney and pancreas TBARS levels. In addition, serum SOD activity and liver and kidney catalase activities were increased with curcumin administration, while heart and pancreas SOD and glutathione-S-transferase activities were reduced [29].

Treatment of STZ-induced, diabetic, Sprague–Dawley rats with curcumin (50 mg/kg b.w./day) for 6 weeks attenuated diabetic nephropathy with significantly reduced blood urea nitrogen, and creatinine while albumin was increased [30]. Kidney heat shock protein (HSP)-27 and mitogen-activated protein kinase (MAPK) p38 protein levels were reduced with curcumin treatment [30]. These data suggest that curcumin’s regulation of diabetic nephropathy may involve changes in post-translational modifications of histone H3 in the kidney. 

Pre-treatment of C57/BL6J mice with curcumin (7.5 mg/kg b.w./day) for 10 h prevented the progression of diabetes induced by multiple low doses of STZ (40 mg/kg b.w.) [31]. Curcumin pre-treatment significantly reduced serum glucose levels and increased insulin levels, glucose clearance and pancreatic glucose transporter 2 (GLUT2) messenger ribonucleic acid (mRNA) levels. In addition, serum and pancreas pro-inflammatory cytokine (interleukin-1β (IL-1β) and tumor necrosis factor alpha (TNF-α)) levels were decreased with curcumin pre-treatment, indicating reduced inflammation [31].

Administration of STZ-induced diabetic Sprague–Dawley rats with curcumin (150 mg/kg b.w./day) for 1 month resulted in reduced blood glucose levels and body weight [32]. Kidney vasoactive factors (endothelial nitric oxide synthase (eNOS) and endothelin-1), transforming growth factor beta 1 (TGF-β1) and extracellular matrix proteins (fibronectin and extradomain-B-containing fibronectin (EBF)) mRNA levels were reduced with the administration of curcumin [32]. In addition, oxidative stress molecular marker, heme oxygenase-1 (HO-1) mRNA level, nuclear 8-hydroxy-2′-deoxyguanosine (8-OhdG) staining, a marker of oxidative DNA damage, cytosolic nitrotyrosine (NT) staining and mesangial expansion in the kidneys were prevented with curcumin treatment, indicating reduced chronic diabetic kidney complications. This was accompanied with reduced p300 and nuclear factor kappa-light-chain-enhancer of activated B cells (NF-kB) activity, suggesting that the effects of curcumin may be due to p300/NF-kB signaling regulation [32].

Treatment of STZ–nicotinamide-induced diabetic Wistar rats with THC (80 mg/kg b.w./day) and chlorogenic acid (CGA; 5 mg/kg b.w./day) for 45 days resulted in significantly reduced fasting plasma glucose and increased plasma insulin levels to levels similar to control non-diabetic animals [33]. In addition, THC and CGA cotreatment reduced liver and kidney oxidative stress with significantly reduced TBARS and hydroperoxide (HP) levels and increased antioxidant enzymes (SOD, catalase, GPx and glutathione) activities [33]. Diabetic morphological changes were attenuated with THC and CGA cotreatment, resulting in moderate focal sinusoidal congestion, focal granuloma, mild sinusoidal dilation and normal glomeruli [33].

The combined treatment of STZ-induced diabetic Swiss albino mice with curcumin (10 mM; 10 µL/mouse i.p.) and a single injection of bone marrow cell (10^6^ BMCs) for 28 days resulted in improved diabetic changes, with increased serum insulin levels and reduced glucose levels [34]. Pancreatic lipid peroxidation proxy MDA levels and pro-inflammatory cytokines (TNF-α and IL-1β) serum levels were significantly reduced with curcumin treatment and BMC transplantation. In addition, pancreatic antioxidant enzymes (SOD, catalase and GPx) activities were increased [34]. This was associated with reduced islet degradation and improved islet regeneration with increased islet mean area and diameter [34].

In a study by Soetikno et al. [35], treatment of STZ-induced, diabetic, Sprague–Dawley rats with curcumin (100 mg/kg b.w./day) for 8 weeks resulted in reduced kidney histological changes and serum glucose, urea nitrogen and creatinine levels, indicating improve kidney function and structure [35]. Curcumin treatment significantly reduced protein kinase C (PKC)-α and PKC-β1 total and membrane protein levels and p300 mRNA levels in kidney tissue. Furthermore, nicotinamide adenine dinucleotide phosphate (NAD(P)H) oxidase subunits, NADPH oxidase 4 (NOX4) and p67phox protein levels and MDA levels were reduced with curcumin treatment, while antioxidant GPx levels were increased. In addition, curcumin treatment reduced extracellular matrix (ECM) pro-fibrotic TGF-β1, connective tissue growth factor (CTGF), osteopontin (OPN), type IV collagen, fibronectin and fetal liver kinase 1 (Flk-1) protein levels in the kidneys [35]. All these proteins are markers of kidney dysfunction and indicators of diabetic nephropathy. These data indicate that curcumin attenuates diabetic nephropathy.

In another study by Soetikno et al. [36], treatment of STZ-induced, diabetic, Sprague–Dawley rats with curcumin (100 mg/kg b.w./day) for 8 weeks attenuated diabetes nephropathy with reduced serum glucose, urea nitrogen and creatinine levels [36]. Pro-inflammatory cytokine TNF-α and IL-1β mRNA and protein levels and macrophage infiltration in kidney tissue was significantly inhibited with curcumin treatment. In addition, IκBα protein levels were increased, while phosphorylated NF-kB and pro-fibrotic intercellular adhesion molecule 1 (ICAM-1), TGF-β1 and monocyte chemoattractant protein 1 (MCP-1) protein levels were reduced with curcumin treatment [36]. These were accompanied with reduced glomerular and tubular histological changes with reduced segmental sclerosis, glomerular hypertrophy and mesangial expansion, indicating that treatment with curcumin reduces STZ-induced diabetic nephropathy [36]. 

Administration of STZ-induced Sprague–Dawley rats with C66 (1 and 5 mg/kg b.w./day), a synthetic analog of curcumin, for 6 weeks significantly reduced serum TNF-α levels and decreased renal macrophage infiltration, indicating reduced renal inflammation [37]. Kidney iNOS, COX-2, TGF-β and MCP-1 protein levels were also reduced. These anti-inflammatory effects of curcumin treatment resulted in reduced renal histopathological abnormalities induced by STZ, with decreased renal glycogen levels and type IV collagen expression [37].

Administration of STZ-induced diabetic Wistar rats with curcumin (60 and 90 mg/kg b.w./day) for 31 days resulted in significantly reduced serum glucose and triglyceride levels and renal proteinuria [38]. Additionally, hepatic enzyme (AST, ALT and ALP) activities were reduced with curcumin treatment. ALT and AST activities are indicative of liver inflammation, while ALP activities are indicative of biliary obstruction, all of which are elevated in non-alcoholic fatty liver disease (NAFLD), seen in T2DM [38].

Oral administration of STZ-induced diabetic Curl:HEL1 rats with a water soluble curcumin derivative (NCD; 10 mg/kg b.w./day) for 45 days resulted in significantly lower serum glucose levels and increased serum insulin levels [39]. NCD treatment reduced serum total cholesterol, triglyceride, LDL cholesterol levels and increased high-density lipoprotein (HDL) cholesterol levels, indicating reduced hyperlipidemia. In addition, lipid peroxidation marker MDA levels in the pancreas, aorta and liver were reduced with NCD treatment, suggesting reduced oxidative stress [39].

Administration of STZ-induced Sprague-Dawley rats with curcumin analog B06 (0.2 mg/kg b.w./day) for 6 weeks significantly decreased kidney and heart inflammation and structural abnormalities [40]. Pro-inflammatory serum TNF-α and nitrite levels were reduced with B06 treatment. In addition, B06 treatment reduced renal macrophage infiltration and TNF-α, COX-2, TGF-β and MCP-1 levels in the kidney and heart [40]. Attenuation of STZ-induced structural and functional abnormalities in the kidney and heart were also observed with B06 treatment, with reduced kidney glycogen levels and collagen IV expression [40].

In a study by Soetikno et al. [41], administration of STZ-induced, diabetic, Sprague–Dawley rats with curcumin (100 mg/kg b.w./day) for 8 weeks resulted in significantly reduced renal and serum triglyceride levels [41]. In addition, curcumin treatment increased the phosphorylation of AMP-activated protein kinase (AMPK) and reduced renal sterol regulatory element-binding protein-1c (SREBP-1c), TGF-β, vascular endothelial growth factor (VEGF) and ECM type IV collagen and fibronectin protein levels. Curcumin treatment also increased nephrin protein levels in diabetic rats to near-normal levels, suggesting improved renal filtration function [41]. 

Treatment of C57BL/6 mice with C66 (5 mg/kg b.w./second day), an analog of curcumin, for 67 days resulted in reduced serum creatinine, albumin and total protein levels, indicating improved kidney function [42]. Renal fibrosis was reduced with curcumin treatment with reduced glycogen and collagen IV levels and macrophage infiltration. In addition, kidney MAPK signaling proteins extracellular signal–regulated kinase (ERK1/2), c-Jun N-terminal kinase (JNK) and p38 phosphorylating levels, were reduced [42]. Serum angiotensin II (Ang II) levels and kidney angiotensin converting enzyme (ACE) mRNA were significantly reduced with curcumin treatment. The data suggest an inhibition of MAPK-mediated ACE expression by the curcumin analog, C66 [42].

Administration of STZ-induced, diabetic Wistar rats with curcumin (100 mg/kg b.w./day) for 12 weeks significantly reduced kidney inflammation and improved function [43,44]. Curcumin administration reduced kidney weight, urinary protein levels and their glomerulosclerosis index, while the creatinine clearance rate was increased, indicating improved kidney filtration. Renal fibrosis, glycogen levels and pro-fibrotic fibronectin and collagen IV protein levels were reduced with curcumin administration [43]. Renal EMT was mediated by curcumin administration with increased p-cadherin and synaptopodin and reduced fibroblast-specific protein 1 (FSP-1), alpha smooth muscle actin (α-SMA) and snail mRNA and protein levels [44]. Pro-inflammatory IL-6, IL-1β and TNF-α protein and cytokine levels were significantly reduced by curcumin treatment in kidney tissues. In addition, kidney pro-inflammatory receptor toll-like receptor 4 (TLR4) mRNA and protein levels and the phosphorylation of caveolin-1 (Cav-1) (Tyr14) was inhibited with curcumin treatment, suggesting that curcumin regulates inflammation by inhibiting the TLR4/cav-1 signaling pathway [43,44].

Treatment of STZ-induced, diabetic, male Wistar rats with curcumin (100 mg/kg b.w./day) and captopril (50 mg/kg b.w./day) for 6 weeks resulted in reduced glucose, total cholesterol and LDL levels, suggesting reduced hyperlipidemia and hyperglycemia [45]. Kidney function was improved by curcumin treatment with reduced serum creatinine and urea nitrogen levels and kidney weight. In addition, curcumin treatment reduced TNF-α, IL-10 and ACE-1 levels [45].

In a study by Sun et al. [46], treatment of STZ-induced, diabetic, male Wistar rats with curcumin (100 and 200 mg/kg b.w./day) for 8 weeks resulted in reduced diabetic nephropathy with reduced kidney weight and albumin excretion, and increased their creatinine clearance rate [46]. Curcumin treatment significantly increased SOD activity, while MDA content and reactive oxygen species (ROS) production was reduced in kidney tissue, indicating reduced oxidative stress. In addition, apoptosis was reduced through curcumin treatment with an increased B-cell lymphoma 2 (Bcl-2) protein level and decreased BCL2 associated X (Bax) and cleaved poly (ADP-ribose) polymerase (PARP) protein levels and reduced caspase-3 activity [46]. Curcumin treatment significantly reduced cav-1 phosphorylation at tyrosine 14, suggesting that curcumin’s effects are mediated by cav-1 regulation [46]. 

Treatment of STZ-induced, diabetic, Wistar rats with curcumin (1.5 mg/kg b.w./day) for 8 weeks significantly reduced serum cholesterol, triacylglycerol and phospholipid levels, while HDL levels were increased, indicating reduced hyperlipidemia [47]. Curcumin treatment reduced oxidative stress markers, MDA, γ-glutamyltranspeptidase and nitric oxide (NO) levels, and increased SOD and GPx activities. Kidney function markers (urea nitrogen and creatinine) serum levels and kidney morphological changes were reduced with curcumin treatment, with reduced glomerular hypertrophy, sclerosis, tubular lipid deposition and macrophage infiltration [47]. Curcumin treatment also decreased vimentin, desmin, SREBP-1, iNOS and TGF-β1 mRNA levels, indicating reduced kidney damage and oxidative stress. In addition, curcumin treatment increased renal synaptopodin, connexin 43 and erythropoietin mRNA levels, suggesting improved podocyte integrity gene expression [47]. 

Oral administration of STZ-induced, diabetic, Wistar rats with curcumin (100 mg/kg b.w./day) for 8 weeks resulted in a reduced serum glucose levels and increased insulin levels, indicating reduced hyperglycemia [48]. The ratio of red pulp (parenchyma and sinusoids) to white pulp (immune lymphocytes and macrophages) was significantly reduced with curcumin administration, suggesting increased immunological function. Spleen tissue ROS levels, NO production, MDA content and protein carboxylation were reduced with curcumin administration, while spleen SOD, catalase, glutathione, GPx and GSH activities were increased, indicating a reduction in oxidative stress [48]. In addition, spleen pro-inflammatory TNF-α, IL-1β, IL-6, MCP-1, ICAM-1 and vascular cell adhesion protein 1 (VCAM-1) mRNA and protein levels and TNF-α and IL-1β cytokine levels were reduced with curcumin treatment. Splenic nuclear NF-κB, COX-2 and iNOS mRNA and protein levels were reduced, while cytosolic NF-κB mRNA and protein levels were increased with curcumin administration, indicating reduced inflammation [48]. Curcumin administration also significantly reduced diabetic-induced apoptosis with reduced caspase-12, calpain-1, phospho-eukaryotic initiation factor 2 alpha (eIF2α), phospho-JNK, phospho-p38, phospho-p53 and Bax protein levels in spleen tissue and increased Bcl-2 and mitochondrial cytochrome c protein levels in spleen tissues [48].

Pre-treatment of STZ-induced, diabetic, Sprague–Dawley rats with nanosystems encapsulating curcumin (nCurcumin; 50 mg/kg b.w./day) for 28 days resulted in reduced serum glucose levels and increased insulin levels, indicating reduced hyperglycemia [49]. Pancreatic islet cell death was significantly reduced with nCurcumin pre-treatment. In addition, pancreatic, pro-inflammatory cytokine (IL-1α, granulocyte-colony stimulating factor (G-CSF), IL-10, IL-17A, IL-1β, IL-6, TNF-α, IL-4, granulocyte-macrophage colony stimulating factor (GM-CSF), interferon gamma (IFN-γ), IL-2, IL-5, IL-13 and IL-12p70) levels and pancreatic 8-oxo-dG levels were reduced with nCurcumin pre-treatment, suggesting reduced pro-inflammatory cytokine-induced apoptosis [49].

Overall, these studies (Table 1) indicate that curcumin administration in STZ-induced diabetic animals resulted in the restoration of blood glucose and lipid levels, improved islet cell regeneration and reduced diabetic nephropathy. In addition, curcumin administration resulted in antioxidant, anti-inflammatory and improved mitochondrial properties. These results occurred through the regulation of multiple signaling molecules, including significantly reduced ERK1/2, JNK, p38, TGF-β1 and NF-κB protein levels and increased phosphorylated AMPK protein levels.

### 2.2. Alloxan-Induced Diabetes Model

The effects of curcumin administration on alloxan-induced diabetic rats are summarized in Table 2. Treatment of alloxan-induced diabetic Wistar rats with curcumin (0.08 mg/kg b.w./day) or turmeric (1 mg/kg b.w./day) for 21 days resulted in significantly reduced serum glucose and hemoglobin A1c (HbA1c) levels, while hemoglobin levels were increased [50]. Liver and serum TBARS level and sorbitol dehydrogenase (SDH) activity were reduced with curcumin or turmeric treatment, while the antioxidant glutathione level and GPx activity were increased. This suggests that both curcumin and turmeric reduce the conversion of sorbitol to fructose [50].

Administration of alloxan-induced, diabetic, Wistar rats with curcumin analogs (compound 4 and compound 10) for 2 h resulted in reduced blood glucose level, similar to levels achieved by treatment with the antidiabetic drug glipizide [51]. Compound 4 has a different aromatic substitution pattern compared to curcumin, with 3,4-dimethoxyphenol for compound 4 and 4-hydroxy-3-methoxyphenyl for curcumin. In addition, compound 10 has a shorter linker region of five carbon atoms compared to curcumin’s seven carbon atoms [51]. These data suggest that different structural compounds of curcumin may have antidiabetic effects and require further studies.

### 2.3. Genetic Diabetes Model

The effects of curcumin administration on genetic animal models of diabetes are summarized in Table 3. Administration of female genetically diabetic KK-Ay/Ta mice with curcumin (1500 mg/kg b.w./day) for 4 weeks resulted in significantly suppressed serum glucose levels [52,53]. Administration of 137 mg/kg b.w./day curcumin did not have any effect on blood glucose levels, while 620 mg/kg b.w./day partially reduced serum glucose levels [53].

Administration of C57BL/KsJ-*db*/*db* mice with curcumin (0.02% *wt*/*wt*) for 6 weeks resulted in significantly reduced serum glucose and HbA1c levels and an increased serum insulin level and homeostatic model assessment of insulin resistance (HOMA-IR) [54]. Plasma fatty acid, total cholesterol and triglyceride levels were significantly reduced with curcumin treatment, indicating reduced hyperlipidemia. Hepatic glucokinase activity was increased, while glucose 6-phosphatase (G6Pase) and phosphoenolpyruvate carboxykinase (PEPCK) activities were reduced with curcumin treatment [54]. The activities of hepatic fatty acid synthase (FAS), β-oxidation, carnitine palmitoyltransferase (CPT), 3-hydroxy-3-methyl-glutaryl-CoA (HMG-CoA) reductase and acetyl-coenzyme A acetyltransferase (ACAT) were reduced with curcumin treatment, suggesting improved lipid metabolism. In addition, liver CAT and GSH-Px activities and MDA level were reduced with curcumin treatment, suggesting that curcumin treatment normalizes antioxidant enzyme activities [54].

In a study by Lu et al. [55], administration of *db*/*db* mice with curcumin (200 mg/kg b.w./day) for 18 weeks resulted in significantly reduced serum glucose levels, body weight and urinary albumin [55]. Renal structural changes were attenuated with curcumin administration, with reduced glomerular sclerosis, mesangial area expansion, thickening of the basement membrane, ECM deposition, collagen IV and fibronectin protein levels. Curcumin administration inhibited phosphorylated signal transducer and activator of transcription 3 (STAT3) and increased IκB protein levels, suggesting that the effects of curcumin may be mediated through the regulation of STAT3 and IκB expression [55].

Administration of *db*/*db* mice with curcumin (60 mg/kg b.w./day) for 4 weeks resulted in significantly reduced serum glucose levels, attenuating the hyperglycemia [56]. In addition, curcumin administration significantly reduced state 3 O_2_ consumption in kidney and state 4 O_2_ consumption in kidney and liver. Liver ATPase activity and NO synthesis was increased, while liver and kidney TBARS level was reduced with curcumin administration, suggesting reduced oxidative stress [56].

Otsuka–Long–Evans–Tokushima Fatty (OLETF) rats represent a genetic strain of non-insulin-dependent diabetes mellitus, characterized with late onset hyperglycemia and mild obesity [57]. In a study by Kim et al. [58], administration of OLETF rats with curcumin (100 mg/kg b.w./day) for 45 weeks resulted in significantly reduced serum glucose, triglyceride and cholesterol levels, while insulin levels and β cell function (HOMA-β) were increased, indicating reduced hyperlipidemia and hyperglycemia [58]. Renal structural changes were attenuated with curcumin administration, with reduced glomerular hypertrophy and glomerular basement membrane (GBM) thickness. In addition, curcumin administration reduced urinary MDA levels and increased urinary SOD activity and renal nuclear factor erythroid 2–related factor 2 (Nrf2) and HO-1 protein levels, suggesting reduced oxidative stress [58]. Renal phosphorylated AMPK and downstream acetyl-CoA carboxylase (ACC) protein levels were increased with curcumin administration, while SREBP-1, SREBP-2 and adipose differentiation-related protein (ADRP) protein levels were reduced, suggesting reduced fatty acid accumulation and synthesis [58]. 

In a mouse model of uncompensated obesity-related insulin resistance the *db*/*db* mice, administration with curcumin (1.5 g/kg b.w./day) resulted in, significantly reduced serum glucose and HbA1c levels and increased insulin levels [59]. Curcumin administration increased surface marker Ki-67-positive expression, a protein marker associated with increased cell proliferation, and β-cell proliferation. Importantly, the mice administered curcumin had increased lean body mass and lifespan [59].

Administration of C57BL/KsJ *db*/*db* mice with curcumin (200 mg/kg b.w./day) for 16 weeks resulted in inhibited nod-like receptor family pyrin domain containing 3 (NLRP3) inflammasome protein levels [60]. Curcumin treatment was also shown to reduce serum creatinine and urea nitrogen levels, renal hypertrophy, glomerular matrix expansion and collagen IV and fibronectin protein levels, suggesting reduced glomerular damage [60]. In addition, kidney pro-inflammatory IL-1*β* and pro-apoptotic caspase-1 protein levels were significantly reduced with curcumin treatment, indicating reduced diabetes-induced inflammation [60].

Overall, these studies (Table 3) indicated that administration of curcumin in genetic diabetic animals resulted in improved glucose homeostasis and islet cell function and reduced lipid serum levels and diabetic nephropathy. In addition, curcumin administration exerted antioxidant, anti-inflammatory and mitochondrial biogenesis properties, which results in improved animal lifespan/survival. 

### 2.4. Diet-Induced Diabetes Model

Several studies used diet-induced, diabetic animal models. The effects of curcumin administration on diet-induced diabetic animals are presented in Table 4. Administration of high-fat-fed Sprague–Dawley rats with curcuminoids (1 g/100 g dietary intake) for 2 weeks resulted in significantly reduced liver triacylglycerol and cholesterol levels and reduced plasma triacylglycerols in the very-low-density lipoprotein (VLDL) fraction [61]. In addition, hepatic acyl-CoA oxidase activity was significantly increased with curcuminoid treatment. Epididymal adipose tissue weight was reduced with curcuminoid intake, suggesting that curcuminoid treatment reduce dyslipidemia [61].

Supplementation of curcumin (0.5% *wt*/*wt*) for 7 days in male swiss albino rats fed a high-cholesterol (1% dietary intake) diet resulted in decreased plasma triglyceride, LDL and cholesterol levels and increased HDL levels [62]. Curcumin supplementation reduced serum AST and ALT enzymatic activities, suggesting reduced lipid peroxidation [62].

In a study by Shao et al. [63], administration of high-fat-fed C57BL/6J mice with curcumin (0.4% dietary intake) for 2 days per week for 28 weeks suggested reduced insulin resistance and obesity. Additionally, epididymal fat mass and serum glucose levels were reduced, while serum adiponectin levels were increased [63]. Curcumin treatment increased phosphorylated protein kinase B (Akt) (Ser473) and reduced phosphorylated glycogen synthase kinases α/β (GSK3α/β) protein levels in both adipose and liver, suggesting improved insulin signaling [63]. In addition, curcumin administration diminished macrophage infiltration in adipose tissue and reduced NF-κB and phosphorylated JNK protein levels, indicating reduced inflammation. Liver lipogenic carbohydrate-response element-binding protein (ChREBP), SREBP-1 and thioredoxin-interacting protein (TxNIP) mRNA levels were significantly reduced with curcumin administration, suggesting attenuated lipogenesis [63].

Administration of high-fat-fed C57BL/6J mice with curcumin (50 mg/kg b.w./day) for 15 days resulted in significantly reduced serum glucose and insulin levels and HOMA-IR, indicating improve insulin sensitivity [64]. Curcumin administration reduced serum MDA level and skeletal muscle mitochondrial and whole tissue MDA and ROS levels. In addition, skeletal muscle total and nuclear Nrf2 protein levels were increased with curcumin treatment, suggesting that curcumin may exert its antioxidant effects through improved Nrf2 signaling [64].

Administration of high-fat-fed (60% kcals) obese C57BL/6 mice with curcumin (0.5% and 1% *wt*/*wt*) for 8 weeks protected against obesity-induced oxidative stress and mitochondrial dysfunction [65]. Curcumin administration significantly decreased liver mitochondrial state 3 and state 4 oxygen consumption and NO synthesis. Lipid peroxidation marker TBARS levels and oxidative protein carbonyl levels were significantly reduced in both liver and kidney with curcumin administration, suggesting reduced oxidative stress [65].

Supplementation of high-fructose, high-fat (HFHF; 60% kcal fructose and 30% kcal fat)-fed Wistar rats with curcumin (200 mg/kg b.w./day) for 10 weeks resulted in significantly reduced serum glucose and body weight [66]. Curcumin treatment increased antioxidant glutathione, GPx, catalase and total antioxidant serum levels, while MDA and total oxidant serum and liver levels were significantly reduced, indicating reduced oxidative stress. In addition, phosphorylated ERK and p38 protein levels were significantly decreased with curcumin treatment, suggesting that the effects may be mediated by ERK/p38 MAPK signaling [66].

Administration of high-fructose-fed (65% kcal/diet) Wistar rats with curcumin (200 mg/kg b.w./day) for 8 weeks resulted in significantly reduced serum glucose, insulin, leptin and TNF-α levels and HOMA-IR, indicating reduced insulin resistance [67]. Curcumin administration improved the lipid profile with significantly reduced serum triglyceride, cholesterol, uric acid and MDA levels. In addition, antioxidant catalase serum activity was increased, while inflammatory cell infiltration in the liver was significantly reduced with curcumin treatment, indicating reduced oxidative stress and inflammation [67].

In a study by Ding et al. [68], administration of high fat fed (60% kcal fat, 20% kcal protein and 20% kcal carbohydrates) C57BL/6 mice with curcumin (80 mg/kg b.w./day) for 12 weeks resulted in significantly reduced body weight gain and fat accumulation in liver and adipose tissues, while there was an increase serum insulin levels [68]. Lipid accumulation and metabolic genes (adiponectin, lipoprotein lipase (LPL) and uncoupling protein 1 (UCP-1)) mRNA levels were increased, while 3-hydroxy-3-methylglutaryl-CoA reductase (HMGCR), FAS and stearoyl-CoA desaturase-1 (SCD-1) mRNA and protein levels were reduced in brown and white adipose tissue with curcumin administration, suggesting reduced lipid synthesis and improved fatty acid oxidation [68]. In addition, liver cholesterol metabolism genes (SREBP-2, HMGCR, squalene synthase (SS), lanosterol synthase (LSS), 24-dehydrocholesterol reductase (DHCR)24, DHCR7 and sterol-C4-methyl oxidase (Sc4mol)) mRNA levels were significantly reduced with curcumin administration. The tyrosine phosphorylation of liver insulin receptor substrate (IRS)-1, IRS-2 and phosphorylation/activation of AKT were improved with curcumin administration, suggesting improved insulin signaling [68].

Administration of high-fat-fed (10% kcal lard and 10% kcal yolk) C57BL/6 mice with curcumin (50 mg/kg b.w./day) for 12 weeks resulted in reduced serum glucose levels [69]. Adipose ER stress and phosphorylated Inositol-requiring enzyme 1 alpha (IRE1α) and eIF2α protein levels were significantly reduced, while phosphodiesterase 3B (PDE3B) and phosphorylated protein kinase A (PKA) protein levels were increased with curcumin administration. Liver phosphorylated Akt protein levels was increased and the G6Pase-α protein level was reduced, suggesting reduced liver insulin resistance [69]. In addition, adipose glycerol and FFA levels and SREBP-1c and phosphorylated HSL protein levels were reduced with curcumin administration, suggesting reduced adipose lipolysis. Treatment with curcumin reduced liver lipid deposits and prevented PKCε translocation to the cell membrane [69].

Overall, these studies (Table 4) provide evidence that the administration of curcumin in diet-induced diabetic animals resulted in attenuated glucose and lipid serum levels and reduced body weight and lipid accumulation in adipose and liver tissue. In addition, curcumin administration exerted anti-inflammatory and antioxidant effects and improved mitochondrial function. Curcumin administration may exert these effects by the regulation of key signaling pathways, with reduced NF-κB, JNK, ERK, p38 and SREBP-1c and increased phosphorylation of IRS-1/IRS-2 and Akt protein levels in insulin target tissues.

## 3. Biological Effects of Curcumin: Human Studies

The biological effects of curcumin administration to healthy and diabetes patients are summarized and presented in Table 5. In a crossover trial, administration of curcumin (6 g) to healthy participants resulted in significantly increased serum insulin levels at 30 and 60 minutes in response to a 75 g oral glucose tolerance test (OGTT) [70]. Curcumin intake had no effect on serum glucose levels [70]. These data suggest that curcumin administration influences insulin secretion.

In the studies by Sukandar et al. [71] and [72], administration of garlic (200 mg/day) and turmeric extract (200 mg/capsule/day) for 12 weeks to T2DM dyslipidemic patients resulted in significantly reduced body mass index (BMI) and serum glucose and HbA1c levels [71,72]. In addition, serum total cholesterol, LDL and triglyceride levels were reduced, while HDL levels were increased with curcumin administration [71,72].

In a randomized, double-blinded and placebo-controlled study, administration of turmeric (500 mg/capsule/meal/day; each capsule containing 22.1 mg turmeric) for 2 months to individuals with diabetic nephropathy resulted in reduced serum glucose, triglyceride, cholesterol and LDL levels [73]. Proteinuria was significantly reduced with turmeric supplementation. In addition, pro-inflammatory cytokine TGF-β and IL-8 serum levels and urinary IL-8 levels were reduced with turmeric supplementation, indicating reduced diabetes-induced inflammation [73]. It is important to note, that no toxic effects occurred with the consumption of turmeric for 2 months in individuals suffering from diabetic nephropathy [73].

Administration of Meriva® (1000 mg/day), a lecithin delivery system of curcumin (200 mg), for 4 weeks to patients with T2DM resulted in reduced diabetic microangiopathy [74,75]. Meriva supplementation reduced blood flux to the skin at the surface of the foot and reduced the edema score, while the venoarteriolar response and partial pressure of oxygen (PO2) were increased [74,75].

In another randomized, double-blinded, placebo-controlled study, administration of curcumin (750 mg/twice per day; 1500 mg/day) for 9 months to pre-diabetic individuals resulted in reduced glucose, HbA1c and insulin levels and HOMA-IR [76]. Curcumin supplementation improved β-cell function, increased homeostatic model assessment (HOMA-β) and reduced serum C-peptide levels. Additionally, anti-inflammatory cytokine adiponectin serum levels were increased with curcumin supplementation, suggesting reduced inflammation [76]. Importantly, curcumin supplementation had no toxic effects and significantly reduced the progress of diabetes with no pre-diabetic individuals progressing to T2DM [76]. 

Supplementation of overweight T2DM individuals (BMI ≥ 24 and FBG ≥ 7 mmol/L) with curcuminoid mixture (36.06% curcumin, 18.85% DMC and 42.58% BDMC; 300 mg/day) for 3 months resulted in decreased serum glucose, HbA1c levels, and HOMA-IR, suggesting improved glycemic control [77]. In addition, curcuminoid mixture supplementation significantly reduced diabetes-induced hyperlipidemia with reduced serum FFAs (palmitic, stearic, oleic, linoleic, γ-linolenic, saturated, unsaturated and total fatty acid), triglycerides and total cholesterol levels and increased serum lipoprotein lipase (LPL) activity [77].

In a randomized, double-blind, placebo-controlled trial, prediabetic (fasting plasma glucose ≥126 mg/dL) and controlled (diet and exercise) diabetic individuals were administered *Jiangtang Xiaozhi* (3.79 g/thrice/day; containing 13% curcumin) for 16 weeks. *Jiangtang Xiaozhi* administration resulted in a significantly reduced serum insulin level and HOMA-IR index score, indicating reduced insulin resistance [78]. However, glucose levels were not affected by this treatment. In addition, patients administered *Jiangtang Xiaozhi* had increased serum HDL level, while total cholesterol, triglyceride and LDL levels were similar [78]. These data suggest that administration of *Jiangtang Xiaozhi* reduces insulin resistance and improves lipid homeostasis in cases of prediabetes and controlled diabetes.

In a randomized, double-blinded, placebo-controlled, crossover trial, administration of obese (BMI ≥ 30) individuals with curcuminoids (1 g/day) for 30 days resulted in a significantly reduced serum triglyceride level, however no effects were shown on serum LDL, total cholesterol or HDL levels [79].

In another randomized, double-blinded and placebo-controlled clinical study by Chuengsamarn et al. [80], supplementation of curcumin (750 mg/twice per day; 1500 mg/day) to T2DM individuals resulted in significantly reduced serum triglyceride, leptin and insulin levels and HOMA-IR, while adiponectin levels were increased, suggesting reduced insulin resistance and inflammation [80]. Visceral and total body fat levels and waist circumference were reduced in individuals supplemented with curcumin, suggesting reduced obesity. In addition, atherogenic risk factors pulse wave velocity (PWV) Rt and PWV Lt was significantly reduced with curcumin supplementation, indicating reduced arterial stiffness and atherosclerosis [80].

In a randomized, double-blind, placebo-controlled study, administration of curcumin (630 mg/thrice/day) for 12 weeks to patients with metabolic syndrome (as defined by the US National Cholesterol Education Program Adult Treatment Panel III, 2001) resulted in significantly increased serum HDL, reduced LDL cholesterol and total triglyceride levels [81]. However, curcumin administration did not change the serum glucose levels or body weight [81].

In a randomized, open-labeled trial, administration of curcumin (475 mg) and glyburide (5 mg) to T2DM patients resulted in significantly reduced serum glucose levels compared to patients administered only glyburide, suggesting that curcumin improves glycemic control [82]. In addition, curcumin administration reduced the serum lipid profile, with reduced LDL, VLDL and triglyceride levels and increased HDL levels [82].

Administration of curcuminoids (300 mg/day) for 3 months to overweight T2DM individuals resulted in significantly reduced serum adipocyte-fatty acid binding protein (A-FABP) levels, suggesting reduced lipolysis in adipose tissue [83]. Pro-inflammatory cytokines TNF-α and IL-6 serum levels were reduced, while SOD activity was increased in T2DM individuals treated with curcuminoids. Correlational data analysis indicated that serum A-FABP levels were positively correlated to serum glucose, FFAs and c-reactive protein (CRP) levels with curcuminoid supplementation [83]. These data suggest that curcuminoid’s reduction of serum A-FABP levels is associated with reduced T2DM metabolic parameters.

In a randomized, controlled study, administration of curcumin complexed with phosphatidylserine (1600 mg/day) for 30 days to overweight individuals with metabolic syndrome resulted in increased weight loss and reduced body fat percentage, waistline circumference, hip circumference and BMI [84].

Oral administration of curcumin (500 mg/day) for 30 days to T2DM patients resulted in significantly reduced urinary-mAlb excretion, suggesting reduced diabetic kidney disease (DKD) progression [85]. Lipid peroxidation index marker MDA plasma levels were significantly reduced, while anti-oxidative serum Nrf2, NAD(P)H:quinone oxidoreductase 1 (NQO-1), SOD1 and SOD2 protein levels were increased in individuals administered curcumin. In addition, individuals administered curcumin had significantly reduced renal microalbumin excretion, and lipopolysaccharide (LPS) content. Furthermore, curcumin increased several gut bacteria (bacteroides, bifidobacterium and lactobacillus) mRNA levels, suggesting improved gut balance and barrier function [85]. Serum lymphocyte pro-inflammatory inhibitory protein, IκB, protein levels were significantly increased, while pro-apoptotic caspase-3 protein levels were reduced [85].

In a randomized, double-blinded clinical trial, administration of nano-micelle curcumin (nano-curcumin; 80 mg/day) for 3 months to T2DM patients resulted in significantly reduced FBG, serum HbA1c, triglyceride and LDL levels [86]. This suggests that nano-curcumin administration to T2DM patients restores lipid profile and glucose homeostasis.

Treatment of patients with diabetes and proteinuric chronic kidney disease (CDK; proteinuria > 1 g protein/day) with curcumin (320 mg/day) for 8 weeks resulted in attenuated lipid peroxidation and enhanced antioxidant capacity by increased serum antioxidant Gpx and SOD activities [87].

In a randomized, double-blinded and placebo-controlled study by Panahi et al. [88], administration of curcuminoids (1000 mg/day) and piperine (10 mg/day) for 12 weeks to T2DM patients resulted in significantly reduced hyperlipidemia [88]. Curcuminoid and piperine administration reduced serum triglyceride, non-HDL cholesterol and lipoprotein(a) levels and increased HDL levels [88]. Curcuminoid (500 mg/day) and piperine (5 mg/day) administration resulted in significantly reduced serum glucose, insulin, creatinine, C-peptide and HbA1c levels and increased HOMA-IR [89]. Additionally, serum ALT and AST levels were decreased with curcuminoid and piperine administration, suggesting reduced hepatic damage [89].

In a randomized, double-blind, parallel group, placebo-controlled trial, administration of a nutraceutical containing *Curcuma longa* (125 mg), *Lagerstroemia speciose* extract (250 mg), *Berberis aristata* extract (155 mg), Alpha-lipoic acid (110 mg), Chrome picolinate (1.3 mg) and Folic acid (0.15 mg) twice per day for 8 weeks to patients with fasting hyperglycemia resulted in improved lipid homeostasis with a tendency to reduce blood glucose levels [90]. Individuals administered the combined nutraceutical had significantly reduced serum triglyceride and increased serum HDL levels. In addition, the fasting insulin level and HOMA-IR index score were significantly reduced with combined nutraceutical administration, suggesting reduced insulin resistance [90].

In a randomized, double-blind, placebo-controlled trial, administration of curcumin (1500 mg/thrice/day) for 10 weeks to T2DM patients resulted in significantly reduced serum glucose levels, suggesting improved glucose homeostasis [91]. In addition, curcumin administration reduced mean weight, BMI and waist circumference [91].

Overall, these clinical studies (Table 5) indicate that administration of curcumin to patients with prediabetes and diabetes improved glucose and lipid homeostasis and improved beta cell function and reduced the progression of diabetes. In addition, kidney and liver function and antioxidant activity were improved with curcumin administration. These studies suggest that curcumin has strong antidiabetic potential. Further studies are required to determine the effective clinical dosage and temporal administration of curcumin.

## 4. Conclusions and Future Directions

**Figure 2:**
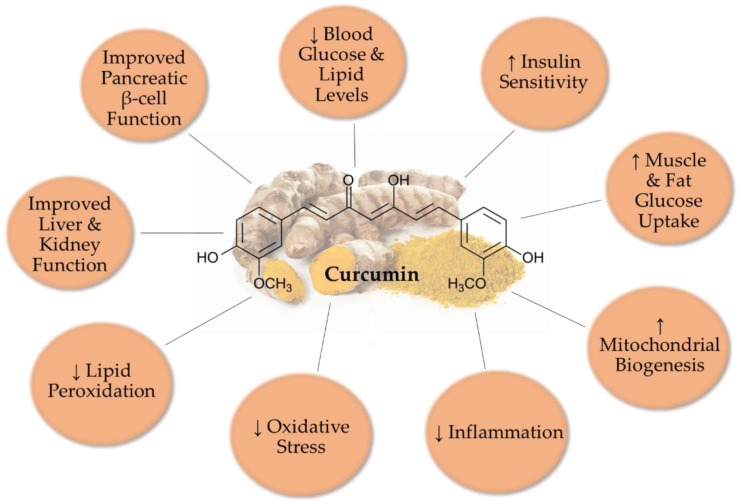
Overall effects of curcumin in T2DM animal models.

Overall, all available in vivo animal studies examining the effects of curcumin indicate significant improved glucose and lipid homeostasis (Figure 2). Serum glucose and lipid levels were significantly reduced. Oxidative stress and lipid peroxidation were reduced with curcumin treatment, while antioxidant enzyme activities were increased. In addition, pro-inflammatory cytokine levels and macrophage infiltration to adipose and liver tissues were reduced. Furthermore, mitochondrial biogenesis was improved with curcumin administration. Administration of curcumin to animal models of diabetic nephropathy resulted in improved kidney function.

The in vivo studies presented in the current review may have used different curcumin dosages and different treatment times. A careful examination of the animal studies revealed that overall, in STZ-induced, diet-induced and genetic models of diabetes, the common doses of curcumin were 100–300 mg/kg b.w./day for 8 weeks. 

The doses of curcumin in human clinical trials were 200–500 mg/day and the common treatment time around 12 weeks. A number of studies have indicated that the use of different drug delivery systems, such as curcumin loaded nanoparticles, liposomes, cyclodextrin inclusions and microemulsions, can result in an increased bioavailability of curcumin and improved action [92,93,94]. We recognize and propose that more human studies should be performed to investigate and establish the effective dose of curcumin. In addition, the detailed effects of curcumin administration on plasma glucose, lipid, insulin and HbA1c levels should be explored.

Many other polyphenols and natural compounds such as resveratrol, naringenin, cinnamon, capsaicin, berberine, genistein and others have been shown to have antidiabetic properties [92,93]. In addition, a systematic and meta-analysis review by Bolignano et al. [95], found that kidney damage due to diabetes is delayed by antioxidant supplementation [95]. Although increased antioxidant intake has been traditionally thought to result in health benefits [96,97], this notion has been challenged lately [98,99], and recent evidence indicates that excess antioxidant intake may increase the risk of certain diseases. Therefore, agents with antioxidant potential, including curcumin, should be studied extensively before recommendations for human supplementation are approved.

The limited human studies indicate that curcumin administration can improve glucose homeostasis and reduce the diabetic phenotype with reduced blood glucose levels and reduced insulin resistance. However, more research must be conducted to fully understand the effects of curcumin in specific tissues of the body, particularly skeletal muscle, adipose tissue, liver and pancreatic β-cells. Overall, the health benefits of curcumin are widespread, and the low toxicity of the molecule makes it a prime candidate for medicinal use against insulin resistance and T2DM. Clearly, more human studies are required to explore the antidiabetic potential of curcumin and curcumin analogs.

## Figures and Tables

**Table 1:** Evidence of antidiabetic effects of curcumin: in vivo Streptozotocin (STZ)-induced diabetes animal studies.

Animal	Curcumin Concentration/Duration	Serum Effects	Other Effects	Reference
Albino Wistar rats	0.5% of diet; 8 weeks	↓ Phospholipid ↓ Triglyceride ↑ PUFA/SFA ratio	↓ Kidney weight ↓ Renal lesion progression ↓ Renal damage ↓ Urine ALT and AST ↓ Kidney alkaline and acid phosphatase ↓ Glucose-6- phosphatase ↓ Lactate dehydrogenase ↑ ATPase activity	[25]
Albino Wistar rats	300 mg/kg b.w./day; 8 weeks	↓ Glucose ↓ Cholesterol ↓ Triglyceride ↓ Urea ↑ Creatinine	↓ Body weight ↓ Kidney lipid peroxidation ↑ Kidney Creatinine excreted ↑ Kidney SOD activity ↑ Kidney Catalase activity	[26]
Wistar Rats	80 mg/kg b.w./day; 45 days	↓ Glucose ↑ Insulin	↓ Lipid peroxidation Kidney: ↑ SOD ↑ Catalase ↑ GPx activity ↑ Glutathione-S-transferase Kidney and liver: ↓ TBARS ↓ H_2_O_2_	[27]
Sprague–Dawley rats	15 and 30 mg/kg b.w./day; 2 weeks	↓ Glucose ↓ Creatinine	↓ Renal changes ↓ Oxidative stress ↓ Urine albumin ↓ Proteinuria ↑ Creatinine clearance ↓ Lipid peroxidation Kidney: ↓ MDA ↑ SOD activity ↑ Catalase activity	[28]
Wistar-NIN rats	0.01% curcumin; 8 weeks	↓ Glucose ↓ Insulin ↑ SOD activity	↓ TBARS ↑ Pancreas catalase activity ↓ Pancreas SOD activity ↓ Glutathione-S-transferase activity	[29]
Sprague–Dawley rats	50 mg/kg b.w./day; 6 weeks	↓ Urea ↓ Creatinine	↑ Albumin ↓ HSP-27 protein ↓ p38 protein ↑ Acetyl-histone H3 ↑ Phospho-histone H3	[30]
C57/BL6J mice	7.5 mg/kg b.w./day; 10 h prior to STZ	↓ Glucose ↑ Insulin ↓ IL-16 ↓ TNF-α	↑ Glucose clearance ↑ GLUT2 mRNA ↓ Pancreatic IL-6 and TNF-α	[31]
Sprague-Dawley rats	150 mg/kg b.w./day; 1 month	↓ Glucose	↓ Body weight ↓ eNOS ↓ TGF-β1 mRNA ↓ Endothelin-1 mRNA ↓ Fibronectin mRNA ↓ EBF mRNA ↓ Oxidative stress ↓ HO-1 mRNA ↓ Nuclear 8-OHdG ↓ Cytosolic NT ↓ Mesangial expansion ↓ p300 activity ↓ NF-κB activity	[32]
Wistar rats	80 mg/kg b.w./day; 45 days	↓ Glucose ↑ Insulin	Kidney and liver: ↓ Morphological changes ↓ Oxidative stress ↓ TBARS ↓ HP ↑ SOD activity ↑ CAT activity ↑ GPx activity ↑ Glutathione activity	[33]
Swiss albino mice	10 mM; 10 µL/mouse i.p.; 28 days and 10^6^ BMCs, single injection	↓ Glucose ↑ Insulin ↓ TNF-α ↓ IL-1β	↑ Islet regeneration Pancreas: ↓ MDA levels ↑ SOD activity ↑ Catalase activity ↑ GPx activity	[34]
Sprague–Dawley rats	100 mg/kg b.w./day; 8 weeks	↓ Glucose ↓ Urea ↓ Creatinine	Kidney: ↓ Histological changes ↓ PKC-α total and membrane protein ↓ PKC-β1 total and membrane protein ↓ P300 mRNA levels ↓ NOX4 and p67phox protein ↓ MDA levels ↑ GPx levels ↓ TGF-β1, VEGF, CTGF and OPN protein ↓ Type IV collagen, fibronectin and Flk-1	[35]
Sprague–Dawley rats	100 mg/kg b.w./day; 8 weeks	↓ Glucose ↓ Urea ↓ Creatinine	Kidney: ↓ Glomerular and tubular histological changes ↓ Segmental sclerosis ↓ Macrophage infiltration ↓ TNF-α and IL-1β mRNA and protein ↑ IκBα protein ↓ ICAM-1, MCP-1 and TGF-β1 protein ↓ Phospho-NF-κB	[36]
Sprague–Dawley rats	1 and 5 mg/kg b.w./day; 6 weeks	↓ TNF-α ↓ Creatinine	Kidney: ↓ Fibrosis ↓ TNF-α mRNA ↓ iNOS mRNA ↓ COX-2 mRNA ↓ TGF-β mRNA ↓ MCP-1 mRNA ↓ Glycogen levels ↓ Type IV collagen expression	[37]
Wistar rats	60 and 90 mg/kg b.w./day; 31 days	↓ Glucose ↓ Triglyceride	↓ Proteinuria ↓ Hepatic AST activity ↓ Hepatic ALT activity ↓ Hepatic ALP activity	[38]
Curl:HEL1 rats	10 mg/kg b.w./day; 45 days	↓ Glucose ↑ Insulin ↓ Cholesterol ↓ Triglyceride ↓ LDL ↑ HDL	↓ Pancreas, aorta and liver MDA levels	[39]
Sprague–Dawley rats	0.2 mg/kg b.w./day; 6 weeks	↓ TNF-α ↓ Creatinine ↓ Nitrite	↓ Renal macrophage infiltration Kidney and cardiac: ↓ TNF-α, COX-2, TGF-β and MCP-1 ↓ Glycogen ↓ Collagen IV expression	[40]
Sprague–Dawley rats	100 mg/kg b.w./day; 8 weeks	↓ Triglyceride	Kidney: ↓ Renal triglyceride ↑ Phospho-AMPK ↓ SREBP-1c protein ↓ TGF-β protein ↓ EGF protein ↓ Type IV collagen protein ↓ Fibronectin protein ↑ Nephrin protein	[41]
C57BL/6 mice	5 mg/kg b.w./second day; 67 days	↓ Creatinine ↓ Albumin ↓ Total protein ↓ Ang II	Kidney: ↓ Fibrosis ↓ Macrophage infiltration ↓ Glycogen levels ↓ Collagen IV ↓ ACE mRNA ↓ Phospho-ERK ↓ Phospho-p38 ↓ Phospho-JNK	[42]
Wistar rats	100 mg/kg b.w./day; 12 weeks	No measured effects	↓ Renal fibrosis ↓ Kidney weight ↓ IL-6, IL-1β and TNF-α protein ↓ Urinary protein ↓ Glomerulosclerosis index ↓ Fibronectin and collagen IV protein ↓ Glycogen ↓ Phospho-Cav-1 ↓ TLR4 mRNA	[43]
Wistar rats	100 mg/kg b.w./day; 12 weeks	↓ Glucose	↓ Renal fibrosis ↓ Kidney weight ↓ Urinary protein ↓ Glomerulosclerosis index ↑ P-cadherin and synaptopodin ↓ FSP-1, α-SMA and snail ↓ Phospho-Cav-1	[44]
Wistar rats	100 mg/kg b.w./day; 6 weeks	↓ Glucose ↓ Cholesterol ↓ LDL ↓ Creatinine ↓ Urea	↑ Body weight ↓ Kidney weight ↓ Nephropathy ↓ Renal ACE1 level Kidney and sciatic nerve: ↓ TNF-α ↓ IL-10	[45]
Wistar rats	100 and 200 mg/kg b.w./day; 8 weeks	No measured effects	↓ Kidney weight ↓ Albumin excretion ↑ Creatinine clearance rate Kidney: ↑ SOD activity ↓ MDA content ↓ Bax and cleaved PARP protein ↓ Caspase-3 activity ↑ Bcl-2 protein ↑ Phospho-cav-1	[46]
Wistar rats	1.5 mg/kg b.w./day; 8 weeks	↓ Cholesterol ↓ Triacylglycerol ↓ Phospholipid ↑ HDL ↓ Urea ↓ Creatinine	↓ Oxidative stress ↓ MDA content ↓ γ-glutamyltranspeptidase level Kidney: ↓ NO ↑ SOD activity ↑ GPx activity ↓ Kidney morphological changes ↓ Vimentin mRNA ↓ Desmin mRNA ↓ SREBP-1 mRNA ↓ iNOS mRNA ↓ TGF-β1 mRNA ↑ Synaptopodin mRNA ↑ Connexin 43 mRNA ↑ Erythropoietin mRNA	[47]
Wistar rats	100 mg/kg b.w./day; 8 weeks	↓ Glucose ↑ Insulin	↑ Splenic white pulp composition ↓ Splenic red pulp composition Spleen: ↓ ROS ↓ NO production ↓ Protein carboxylation ↑ SOD, catalase, glutathione, GPx and GSH activities ↓ TNF-α, IL-1β, IL-6, MCP-1, ICAM-1 and VCAM-1 ↓ TNF-α and IL-1β ↓ Nuclear NF-κB, COX-2 and iNOS ↑ Cytosolic NF-κB ↓ Caspase-12, calpain-1 and Bax ↑ Bcl-2 and mitochondrial cytochrome c ↓ Phospho-eIF2α, JNK, p38 and p53	[48]
Sprague–Dawley rats	50 mg/kg b.w./day; 28 days	↓ Glucose ↑ Insulin	↓ Islet death Pancreas: ↓ IL-1α, G-CSF, IL-10, IL-17A, IL-1β, IL-6, TNF-α, IL-4, GM-CSF, IFN-γ, IL-2, IL-5, IL-13 and IL-12p70 ↓ 8-oxo-dG	[49]

↓ Reduced effect; ↑ Increased effect.

**Table 2:** Evidence of antidiabetic effects of curcumin: in vivo alloxan-induced diabetes animal studies.

Animal	Curcumin Concentration/Duration	Serum Effects	Other Effects	Reference
Wistar rats	0.08 mg/kg b.w./day; 21 days	↓ Glucose ↓ HbA1c ↑ Hemoglobin ↓ TBARS ↑ Glutathione	Liver: ↓ Liver TBARS ↓ SDH activity ↑ Liver glutathione ↑ GPx activity	[50]
Wistar rats	0.1 mg/kg b.w.; 2 h	↓ Glucose	No measured effects	[51]

↓ Reduced effect; ↑ Increased effect.

**Table 3:** Evidence of the antidiabetic effects of curcumin: in vivo genetic diabetes animal studies.

Animal	Curcumin Concentration/Duration	Serum Effects	Other Effects	Reference
KK-Ay mice	1500 mg/kg b.w./day; 4 weeks	↓ Glucose	No measured effects	[52]
KK-Ay mice	137, 620 and 1500 mg/kg b.w./day; 4 weeks	↓ Glucose	No measured effects	[53]
C57BL/KsJ-*db*/*db* Mice	0.02%, *wt*/*wt*; 6 weeks	↓ Glucose ↓ HbA1C ↑ Insulin ↓ Fatty acid ↓ Cholesterol ↓ Triglyceride	Liver: ↑ Glucokinase activity ↓ G6Pase activity ↓ PEPCK activity ↓ FAS activity ↓ β-oxidation activity ↓ CPT activity ↓ HMG-CoA reductase activity ↓ ACAT activity ↓ CAT activity ↓ GSH-Px activity ↓ MDA level	[54]
*db*/*db* mice	200 mg/kg/day; 18 weeks	↓ Glucose	↓ Body weight Kidney: ↓ Albuminuria ↓ Glomerular sclerosis ↓ Mesangial area expansion ↓ Thickening of membrane ↓ ECM deposition ↓ Phospho-STAT3 ↑ IκB protein	[55]
*db*/*db* mice	60 mg/kg/day; 4 weeks	↓ Glucose	Kidney and liver: ↓ Mitochondrial dysfunction ↓ State 3 O_2_ consumption ↓ State 4 O_2_ consumption ↓ TBARS level Liver: ↑ ATPase activity ↑ NO synthesis	[56]
OLETF rats	100 mg/kg/day; 45 weeks	↓ Glucose ↓ Triglyceride ↓ Cholesterol ↑ Insulin	↑ β cell function ↓ Glomerular hypertrophy ↓ GMB thickness ↓ Albuminuria Kidney: ↓ MDA ↑ SOD ↑ Nrf2 and HO-1 protein ↑ Phospho-AMPK and ACC ↓ SREBP-1, -2 and ADRP	[58]
Lepr^db/db^ mice	1500 mg/kg b.w./day	↓ Glucose ↓ HbA1c ↑ Insulin	↓ β-cell loss ↑ Ki-67-positive ↑ Insulin production ↑ Lean body mass ↑ Lifespan	[59]
C57BL/KsJ db/db mice	200 mg/kg/day; 16 weeks	↓ Glucose ↓ Creatinine ↓ Urea	↓ Renal hypertrophy ↓ Glomerular matrix ↓ Kidney weight ↓ NLRP3 protein ↓ Collagen IV and fibronectin ↓ IL-1β protein ↓ Caspase-1 protein	[60]

↓ Reduced effect; ↑ Increased effect.

**Table 4:** Evidence of antidiabetic effects of curcumin: in vivo diet-induced diabetes animal studies.

Animal	Curcumin Concentration/Duration	Serum Effects	Other Effects	Reference
Sprague–Dawley rats	1 g/100 g diet; 2 weeks	↓ Triacylglycerol	↓ Hepatic triacylglycerol levels ↑ Hepatic acyl-CoA oxidase ↓ Epididymal adipose tissue wt	[61]
Male swiss albino rats	0.5% *wt*/*wt*; 7 days	↓ Triglyceride ↓ Cholesterol ↓ LDL ↑ HDL ↓ AST activity ↓ ALT activity	No measured effects	[62]
C57BL/6J Mice	0.4% dietary intake; 2 days/week for 28 weeks	↓ Glucose ↓ Insulin ↑ Adiponectin	↓ Body weight ↓ Epididymal fat mass ↓ Liver lipogenesis ↑ Liver and adipose phospho-Akt ↓ Liver and adipose phospho-GSKα/β ↓ Macrophage infiltration ↓ NF-κB protein ↓ Phospho-JNK ↓ ChREBP, SREBP-1 and TxNIP	[63]
C57BL/6J mice	50 mg/kg b.w./day; 15 days	↓ Glucose ↓ Insulin ↓ MDA	↓ HOMA-IR Skeletal muscle: ↓ Mitochondrial MDA levels ↓ Mitochondrial ROS levels ↑ Nrf2 protein levels ↑ Nuclear Nrf2 protein levels	[64]
C57BL/6 mice	0.5% and 1% *wt*/*wt*; 8 weeks	↓ Triglyceride	↓ Oxidative stress ↓ Mitochondrial dysfunction ↓ Oxygen consumption Kidney: ↑ State 3 O_2_ consumption ↑ State 4 O_2_ consumption ↑ mitochondrial NO synthesis ↓ TBARS levels Liver: ↓ Protein carbonyl ↓ TBARS levels	[65]
Wistar rats	200 mg/kg b.w./day; 10 weeks	↓ Glucose ↑ Glutathione ↑ GPx ↑ Catalase ↓ MDA ↓ Total oxidant	↓ Body weight ↓ Oxidative stress Liver: ↓ MDA levels ↓ Total oxidant levels ↑ Total antioxidant levels ↓ Phospho-ERK ↓ Phospho-p38	[66]
Wistar rats	200 mg/kg b.w./day; 8 weeks	↓ Glucose ↓ Insulin ↓ TNF-α ↓ Leptin ↓ Triglyceride ↓ Cholesterol ↓ Uric acid ↓ MDA ↑ Catalase	↓ HOMA-IR ↓ Oxidative stress ↓ Liver inflammation	[67]
C57BL/6 mice	80 mg/kg b.w./day; 12 weeks	↓ Insulin ↓ Triglyceride ↓ Cholesterol ↓ LDL	↓ Body weight gain Liver: ↑ Insulin sensitivity ↓ Fat accumulation ↑ Adiponectin, LPL and UCP-1 ↓ HMGCR, FAS and SCD-1 ↑ Phospho-IRS-1 ↑ Phospho-IRS-2 ↑ Phospho-AKT	[68]
C57BL/6 mice	50 mg/kg b.w./day; 12 weeks	↓ Glucose	↓ Adipose glycerol and FFA ↓ Liver lipid deposits Adipose: ↓ ER stress ↓ Phospho-IRE1α ↓ Phospho-eIF2α ↑ PDE3B protein ↑ Phospho-PKA ↑ Phospho-Akt ↓ G6Pase-α protein ↓ SREBP-1c protein	[69]

↓ Reduced effect; ↑ Increased effect.

**Table 5:** Effects of curcumin: Human studies.

Condition	Curcumin Concentration/Duration	Serum Effects	Other Effects	Reference
Healthy individuals	6 g; 30 and 60 mins	↑ Insulin	No additional effects	[70]
T2DM dyslipidemia patients	200 mg/capsule/day; 12 weeks	↓ Glucose ↓ Triglyceride ↓ LDL ↑ HDL	↓ BMI	[71]
T2DM patients	200 mg/day; 14 weeks	↓ Glucose ↓ HbA1c	No additional effects	[72]
Diabetic Nephropathy	22.1 mg/day; 2 months	↓ Glucose ↓ Triglyceride ↓ Cholesterol ↓ LDL ↓ TGF-β ↓ IL-8	↓ Proteinuria ↓ Urinary IL-8	[73]
Diabetic Patients	200 mg/day; 4 weeks	No measured effects	↓ Microangiopathy ↑ Venoarteriolar response ↑ PO2 ↓ Skin flux ↓ Edema	[74]
Diabetic Patients	200 mg/day; 4 weeks	No measured effects	↓ Microangiopathy ↑ Venoarteriolar response ↑ PO2 ↓ Skin flux ↓ Edema	[75]
Pre-Diabetic Patients	1500 mg/day; 9 months	↓ Glucose ↓ HbA1c ↓ Insulin ↓ C-peptide ↑ Adiponectin	↓ Diabetes ↑ β-cells function ↑ HOMA-β	[76]
Overweight Diabetic Patients	300 mg/day; 3 months	↓ Glucose ↓ HbA1c ↓ Triglycerides ↓ Total cholesterol ↓ Total fatty acid ↓ Saturated FFA ↓ Unsaturated FFA ↑ LPL activity	↓ HOMA-IR	[77]
Pre-diabetic and controlled diabetic patients	3.17 g *Jiangtang Xiaozhi* (13% curcumin); 16 weeks	↓ Insulin ↑ HDL	↓ HOMA-IR	[78]
Obese patients	1 g/day; 30 days	↓ Triglyceride	No additional effects	[79]
Diabetic patients	1500 mg/day; 6 months	↓ Insulin ↓ Triglyceride ↓ Leptin ↑ Adiponectin	↓ HOMA-IR ↓ Visceral fat ↓ Total body fat ↓ Waist ↓ PWV Rt ↓ PWV Lt	[80]
Metabolic syndrome patients	630 mg/thrice/day; 12 weeks	↑ HDL ↓ LDL ↓ Total triglyceride	No additional effects	[81]
T2DM patients	475 mg/once; 24 h	↓ Glucose ↓ Triglyceride ↓ LDL ↓ VLDL ↑ HDL	No additional effects	[82]
Overweight Diabetic patients	300mg/day; 3 months	↓ A-FABP ↓ CRP ↓ TNF-α ↓ IL-6 ↑ SOD activity	No measured effects	[83]
Overweight patients with metabolic syndrome	1600 mg/day; 30 days	No measurements	↑ Weight loss ↓ Body fat % ↓ Waist ↓ Hip circumference ↓ BMI	[84]
Diabetic patients	500 mg/day; 15-30 days	↓ MDA ↓ LPS content ↓ U-mAlb ↑ IκB ↑ NQO-1 ↑ Nrf2 ↑ SOD1/2 ↓ Caspase-3 ↑ Bacteroides ↑ Bifidobacterium ↑ Lactobacillus	No measured effects	[85]
T2DM patients	80 mg/day; 3 months	↓ Fasting glucose ↓ HbA1c ↓ Triglyceride ↓ LDL	↓ BMI	[86]
Diabetic proteinuric CKD patients	320 mg/day; 8 weeks	↑ Gpx activity ↑ SOD activity	↓ Lipid peroxidation	[87]
Diabetic patients	1000mg/day; 12 weeks	↓ Triglyceride ↓ Non-HDL ↓ Lipoprotein ↑ HDL	No measured effects	[88]
Diabetic patients	500 mg/day; 3 months	↓ Glucose ↓ HbA1c ↓ Insulin ↓ C-peptide ↓ Creatinine ↓ ALT and AST	↑ HOMA-IR ↓ Hepatic damage	[89]
Fasting glucose impaired patients	125 mg/twice/day; 8 weeks	↓ Fasting insulin ↓ Triglycerides ↑ HDL	↓ HOMA-IR	[90]
T2DM patients	1500 mg/thrice/day; 10 weeks	↓ Glucose	↓ Mean weight ↓ BMI ↓ Waist	[91]

↓ Reduced effect; ↑ Increased effect.
